# Dietary *Pieris japonica* Leaf Powder Enhances Hepatic Antioxidant Capacity, Modulates Cecal Microbiota, and Improves Eggshell Thickness in Laying Hens

**DOI:** 10.3390/vetsci13101116

**Published:** 2026-10-19

**Authors:** Yongmei Wu, Jiayi Chen, Fengming Chen, Yang Liu, Jingyan Lian, Yune Tang, Binsheng He, Qian Lin

**Affiliations:** 1 Hunan Provincial Key Laboratory of the Traditional Chinese Medicine Agricultural Biogenomics, Changsha Medical University, Changsha 410219, China; wu@csmu.edu.cn (Y.W.); jiayi@csmu.edu.cn (J.C.); cfming@csmu.edu.cn (F.C.); liuyang0305@hunaas.cn (Y.L.); 20262023211614@stu.usc.edu.cn (J.L.); tangyune@hunnu.edu.cn (Y.T.); 2 Key Laboratory of Regional Characteristic Agricultural Resources, Neijiang Normal University, Neijiang 641100, China; 3 Institute of Bast Fiber Crops, Chinese Academy of Agricultural Sciences, Changsha 410017, China

**Keywords:** *Pieris japonica*, egg quality, antioxidant capacity, intestinal health

## Abstract

Oxidative stress and intestinal health are critical factors that are closely linked to the production performance and physiological status of laying hens. This work assessed how dietary incorporation of *Pieris japonica* leaf powder (PJP) shapes egg quality attributes, hepatic antioxidant defence, gut tissue morphology, and cecal microbial community profiles in layers. A total of 360 healthy 56-week-old Hy-Line Brown layers were randomly assigned to four treatments, each containing six replicate pens housing 15 birds apiece. Animals received basal feeds containing 0.00% (control), 0.05%, 0.10%, or 0.20% PJP throughout the 10-week experimental run that including a 2-week adaptation period. Relative to the control group, diets with 0.05% PJP significantly increased eggshell thickness (*p* < 0.05). Hepatic total antioxidant capacity (T-AOC), catalase (CAT), superoxide dismutase (SOD), and glutathione peroxidase (GSH-Px) were significantly elevated in PJP-supplemented groups (*p* < 0.05). The 0.10% PJP addition improved duodenal villus-height-to-crypt-depth ratio (V/C). Regarding cecal microbiota, 0.10% PJP reduced the Simpson index while elevating *Collinsella* relative abundance (*p* < 0.05). Noticeably, the 0.20% supplementation exerted adverse effects on egg-related indices. Correlation analysis indicated that cecal microbiota was closely associated with hepatic antioxidant indices and eggshell-related indicators. In summary, dietary PJP inclusion may improve the eggshell thickness of eggs from laying hens by enhancing host antioxidant defense, reshaping the composition of cecal microbial communities. It was reported that 0.10% PJP supplementation yielded the most favorable comprehensive responses under the conditions adopted within this trial.

## Simple Summary

Late-phase laying hens frequently experience oxidative damage and impaired intestinal health, lowering egg quality and causing economic losses for poultry farmers. The present work set out to characterize how basal diets supplemented with *Pieris japonica* leaf powder (PJP) alter egg-related measurements, hepatic antioxidant status, duodenal histomorphology, and cecal microbial profiles of layers. Over a 10-week animal feeding assay, 56-week-old commercial layers were allocated to four dietary groups receiving graded inclusion rates of PJP. Moderate PJP supplementation increased eggshell thickness, enhanced hepatic antioxidant capacity, improved duodenal mucosal morphology, and reshaped cecal bacterial communities. High-dose PJP exerted adverse effects on egg-related indices. Among the tested dosages, the 0.10% inclusion level produced the most favorable comprehensive outcomes. This plant powder exhibits potential for development as a plant-derived feed additive candidate, while its feeding safety still requires further validation before practical application in commercial egg-production farms.

## 1. Introduction

Late-phase laying hens commonly experience accumulated oxidative damage and impaired physiological status after long-term egg production [1]. Accordingly, natural botanical feed additives possessing free-radical scavenging and anti-inflammatory bioactivities are gaining growing attention as viable nutritional strategies [2,3]. Dietary supplementation with such plant-based additives has been reported to modulate cecal microbiota [4], enhance antioxidant and immune responses [5], and improve intestinal structure and egg-related indices in laying hens, as exemplified by an Allium-based phytobiotic [6] and by dietary garcinol during the late laying stage [7]. Furthermore, the cecal microbiota of laying hens undergoes dynamic, stage-specific shifts from hatching to the late laying period, and specific microbial taxa are closely associated with growth performance and intestinal health [8], underscoring cecal microbial homeostasis as a critical determinant of egg production and egg-related traits. Nevertheless, although these findings collectively demonstrate the general benefits of plant-derived additives in poultry, systematic investigations into most individual plant species remain scarce, and their applicability to late-phase laying-hen production constitutes a clear knowledge gap.

*Pieris japonica* (*P. japonica*), a member of the Ericaceae family, exhibits multiple biological activities, including antioxidant [9], anti-inflammatory [10], and analgesic [11] properties. Previous in vitro work showed that *P. japonica* leaf material is rich in flavonoids and possesses strong antioxidant and anti-inflammatory capacity, with an ABTS free-radical-scavenging rate of 85.74% [9], suggesting its potential to be developed as a plant-derived feed material for poultry. However, to our current knowledge, no controlled feeding work has characterized the physiological responses triggered by dietary *P. japonica* for laying hens; suitable inclusion rates for this botanical material still remain undefined. Therefore, based on its reported bioactivities, we hypothesized that adding *Pieris japonica* leaf powder (PJP) into basal feeds would enhance productive performance, egg quality, serum biochemical profiles, hepatic antioxidant capacity, intestinal morphology and sustain cecal microbial homeostasis among late-cycle layers, and that response magnitudes would vary according to supplemental dosage.

## 2. Materials and Methods

### 2.1. Preparation of PJP

All animal-related experimental procedures were reviewed and approved by the Science and Technology Ethics Review Committee of Changsha Medical University, Changsha, China (Approval No. CSMUX2023202; approval date: 25 November 2023).

Dried leaves of *P. japonica* were obtained from Kunming Caizhi Biotechnology Co., Ltd. (Kunming, China) and pulverized using a sealed pulverizer (AF-20S, Wenling Aoli Traditional Chinese Medicine Machinery Co., Ltd., Wenling, China), then passed through an 80-mesh screen to obtain a fine powder. To ensure stability, the prepared powder was sealed in light-resistant packaging and maintained at room temperature (approximately 20–25 °C) in a dark storage area until it was incorporated into the experimental diets. The nutritional composition of *P. japonica* included crude protein (CP) at 8.25%, crude ash at 3.71%, crude fat at 3.74%, calcium at 2.28%, phosphorus at 0.03%, and an energy content of 20.35 kJ/g.

### 2.2. Animals and Experimental Details

This trial adopted a single-factor experimental design and utilized 360 clinically healthy 56-week-old Hy-Line Brown layers. Birds were randomly allocated into four dietary treatment groups, with six replicate pens per treatment holding 15 hens each. The control group was fed exclusively the basal diet, whereas the three-treatment group received basal feeds supplemented with 0.05%, 0.10%, and 0.20% PJP, respectively. The entire experimental period spanned 10 weeks, encompassing a 2-week pre-experimental preliminary phase. Table 1 summarizes the ingredient profile and respective nutrient contents of the basal experimental diet. Since no published poultry feeding studies are available for PJP, the three inclusion levels (0.05%, 0.10%, and 0.20%) were selected by referring to the typical dosage ranges of other medicinal-plant powder additives for laying hens (0.04–0.20%) [12,13]. To achieve even dispersion at these low supplementation rates, every PJP dosage was initially combined with a minor portion of basal feed to generate a concentrated premix. This premix was subsequently incorporated incrementally into the full basal ration via serial dilution; every formulated diet underwent 10 min of mechanical mixing prior to animal feeding. The maximum level of 0.20% was further verified in the 2-week preliminary phase, during which no adverse clinical responses were observed.

### 2.3. Feeding Management and Sample Collection

All animal procedures took place within the authorized animal experimental premises affiliated with Changsha Medical University. Vaccination procedures complied with standard disease-preventive guidelines [14] adopted within commercial large-scale poultry production systems. Replication for this trial was arranged following a rigorous three-level experimental design. Birds received two daily feed allocations at 07:00 and 15:30, with drinking water continuously supplied through nipple drinkers. The photoperiod was set to 16 h of light each day. Room temperature was stabilized at 25 ± 2 °C. Relative humidity was held at roughly 65% throughout the whole feeding phase. Egg harvesting was performed each day at 08:00 and 17:00 to carry out subsequent egg-quality assessments. Daily documentation was kept regarding egg counts, individual egg weights, as well as daily feed consumption.

Upon completion of the feeding trial, two birds per pen were picked at random for venous blood harvesting. This sampling scheme yielded a total of 12 biological replicates for every dietary group. Whole-blood aliquots were permitted to clot under ambient conditions for roughly 30 min, prior to centrifugation running at 3500 rpm over a 15 min period. Isolated serum fractions were preserved at −20 °C for later biochemical measurements. Following blood collection, euthanasia was conducted using carbon dioxide inhalation via a specialized laboratory animal euthanasia system (Model KW-AL-G, Nanjing Calvin Biotechnology Co., Ltd., Nanjing, China) with a 100% CO_2_ concentration. Loss of vital signs, including halted breathing and non-detectable heartbeat lasting more than 5 min, served as confirmation of death. Once the abdominal cavity was opened, 3–5 cm tissue fragments from the duodenum, jejunum and ileum were excised and fixed within 4% neutral-buffered formalin solution. Cecal digesta were retrieved and kept at −80 °C to perform downstream cecal microbiota profiling. Simultaneously, liver aliquots weighing 2 g were obtained and archived at −20 °C for follow-up antioxidant biomarker quantification.

### 2.4. Production Performance

Egg counts as well as cracked-egg quantities for each replicate unit were logged each day. Daily feed consumption together with individual egg mass were quantified with an electronic weighing balance. Laying rate was defined as the proportion of laid eggs against the total hen population within each experimental unit. Cracked-egg proportion was derived by dividing the count of defective shelled eggs by the overall egg yield. The feed conversion ratio (FCR) was computed every week over the 8-week formal experimental period (excluding the 2-week adaptation phase) and expressed as g feed/g egg. All computational operations were performed at the pen-level replicate: cumulative weekly feed consumption from each pen was normalized against aggregate weekly egg mass generated by that same housing unit. Mortality events were tracked and documented on a daily basis for the full duration of the trial.

### 2.5. Egg Quality Measurements

At the experimental endpoint, egg-quality assessments were carried out utilizing 12 eggs sourced from every replicate pen. An analytical balance (ME204, Mettler Toledo, Greifensee, Switzerland) was applied to record egg mass and yolk mass. Egg shape index was obtained via the standard formula: (Egg Width/Egg Length) × 100. A digital electronic vernier caliper (500-196-30, Mitutoyo Corporation, Kanagawa, Japan) was used to acquire egg width and egg-length readings. Haugh unit values alongside yolk color scores were determined with the aid of an egg quality analyzer (EA-01, ORKA Food Technology Ltd., Ramat Hasharon, Israel). Eggshell thickness, which represents the mean value derived from readings collected at the sharp end, equator, and blunt end of each egg, was quantified with a digital eggshell thickness gauge (HADMA3, Beijing Hengdao Instrument Co., Ltd., Beijing, China).

### 2.6. Serum Biochemical Indices

A series of serum biochemical parameters were quantified to assess systemic physiological status. Serum biochemical parameters, including total protein (TP), albumin (ALB), globulin (GLB), blood urea nitrogen (BUN), aspartate aminotransferase (AST), alanine aminotransferase (ALT), alkaline phosphatase (ALP), lactate dehydrogenase (LDH), glucose (GLU), total cholesterol (TC), triglycerides (TGs), high-density lipoprotein (HDL), and low-density lipoprotein (LDL), were quantified using commercial assay kits purchased from Nanjing Jiancheng Bioengineering Institute (Nanjing, China). All measurements were performed strictly according to the standard operating protocols provided by the kit manufacturer. Sample readings were acquired on a Mindray BS-200 automated biochemistry analyzer (Shenzhen Mindray Biomedical Electronics Co., Ltd., Shenzhen, China), in accordance with operational specifications specified within the corresponding commercial assay kits (Nanjing Jiancheng Bioengineering Institute, Nanjing, China).

### 2.7. Antioxidant Indices Determination

The antioxidant profile of liver tissue was assessed by measuring levels of glutathione (GSH), catalase (CAT), superoxide dismutase (SOD), glutathione peroxidase (GSH-Px), total antioxidant capacity (T-AOC), and the lipid peroxidation product malondialdehyde (MDA). Commercial assay kits from Nanjing Jiancheng Bioengineering Institute (Nanjing, China) were used for all determinations, with procedures performed as specified. TP concentration in the hepatic homogenates was determined using the Coomassie Brilliant Blue method.

### 2.8. Intestinal Morphology Analysis

Segments measuring 3–5 cm in length were excised from the duodenum, jejunum and ileum, and immediately fixed in 4% paraformaldehyde solution. Following preservation, the specimens underwent dehydration in alcohol, paraffin embedding, and sectioning for H&E staining. The slides were digitized using the Pannoramic MIDI pathological scanning system (3DHISTECH Ltd., Budapest, Hungary). Villus height (VH) and crypt depth (CD) for every intestinal specimen were determined utilizing CaseViewer software (Version 2.4; 3DHISTECH Ltd., Budapest, Hungary) and their quotient was computed to obtain the villus-height-to-crypt-depth ratio (V/C). For robust statistical outputs, a minimum of 15 independent readings were taken per intestinal sample, and resultant mean values were used for subsequent statistical analysis.

### 2.9. Cecal Microbiome Analysis

Cecal digesta were retrieved and flash-frozen right away in liquid nitrogen, then kept at −80 °C pending downstream profiling. Genomic DNA for microbial communities was isolated with the PowerSoil DNA Isolation Kit (MO BIO Laboratories, Inc., Carlsbad, CA, USA). Purified DNA preparations were checked for integrity and concentration via 1% agarose gel electrophoresis together with spectrophotometric quantification. Target amplification of the V3-V4 hypervariable segment within the 16S rRNA gene was carried out with primer pairs 338F (5′-ACTCCTACGGGAGGCAGCAG-3′) and 806R (5′-GGACTACNNGGGTATCTAAT-3′). Recovered purified amplicons underwent sequencing on the Illumina MiSeq PE300 platform at Personalbio (Shanghai, China). Subsequent bioinformatics processing relied on the standard workflow offered by Personalbio, covering quality trimming, chimera elimination, and amplicon sequence variant (ASV) detection. Taxonomic assignments were completed by referencing the SILVA reference database. QIIME1 (version 1.8.0) software was deployed to compute α-diversity metrics, including Chao1, Observed-species, Pielou, Simpson, Shannon, and Good-coverage. Based on taxonomic annotation and relative abundance results, R software (v3.6.0) was applied to generate bar charts for taxonomic composition. A clustered heatmap was generated within R (v3.6.0) employing Weighted Unifrac distance metrics. The raw amplicon-sequencing-derived ASV feature table, taxonomic annotation table and sample metadata table are provided in Supplementary Materials (Tables S1–S4).

### 2.10. Statistical Analyses

Statistical computations were carried out in SPSS 23.0, with outcomes presented as mean ± standard error (SE). One-way analysis of variance (ANOVA) was implemented across all measured variables. Levene’s test was adopted to verify variance homogeneity. When significant heterogeneity of variance was detected, Welch’s ANOVA was used for statistical comparison. Upon obtaining significant overall ANOVA results, Duncan’s multiple-range test served for post hoc pairwise comparisons between different treatment cohorts. The statistical cutoff for significance was established at *p* < 0.05.

## 3. Results

### 3.1. PJP Maintains Stable Laying Performance in Laying Hens

Statistical analyses indicated that key production indicators remained stable across all feeding regimens, with no significant differences observed (*p* > 0.05, Table 2). Consequently, dietary supplementation of PJP at concentrations ranging from 0.05% to 0.20% exerted no detrimental impacts on laying performance in hens.

### 3.2. 0.05% PJP Increases Eggshell Thickness, Whereas 0.20% PJP Numerically Reduces Egg Quality

The effects of dietary supplementation with PJP on egg quality are presented in Table 3. No statistically significant differences were observed in egg weight, eggshell weight, egg yolk weight, egg white weight, or egg shape index among the control and treatment groups (*p* > 0.05). Relative to the control cohort, diets containing 0.05% PJP markedly elevated eggshell thickness (*p* < 0.01). Yolk color, albumen height and Haugh-unit readings registered greater values within the 0.05% and 0.10% cohorts when compared against the 0.20% group, while these parameters exhibited no statistically detectable divergence from control values. Notably, the highest-dose 0.20% treatment yielded significantly reduced albumen height and Haugh-unit scores versus the 0.05% and 0.10% groups (*p* < 0.05); these two indicators also showed non-significant numerical declines compared with the control treatment.

### 3.3. PJP Increases Hepatic Antioxidant Enzyme Activities and Total Antioxidant Capacity in Laying Hens

Table 4 reveals that dietary PJP inclusion markedly improved the antioxidant status of laying hens. The activities of CAT, SOD, and GSH-Px in the liver showed a marked increase specifically in hens receiving the 0.20% PJP supplement relative to controls (*p* < 0.05). Additionally, hepatic T-AOC was substantially elevated in all PJP-supplemented treatments (*p* < 0.01). Moreover, hepatic MDA levels exhibited a non-significant downward trend across all treatment groups.

### 3.4. PJP Modulates Serum Protein-Related Parameters Without Inducing Hepatic Injury

Table 5 depicts shifts in major serum biochemical markers in laying hens receiving dietary PJP. The incorporation of PJP did not significantly alter the levels of ALT, ALP, GLU, TC, AST, AST/ALT, ALB/GLB, TG, LDH and LDL (*p* > 0.05). In the 0.10% treatment, serum TP, GLB, and ALB concentrations were higher relative to the basal diet control (*p* < 0.05). Dietary PJP addition lowered serum HDL in the 0.05% and 0.20% groups and increased BUN in the 0.10% group, with these differences reaching statistical significance compared with the basal diet group (*p* < 0.05).

### 3.5. PJP Improves Duodenal Intestinal Morphology of Laying Hens

**Figure 1:**
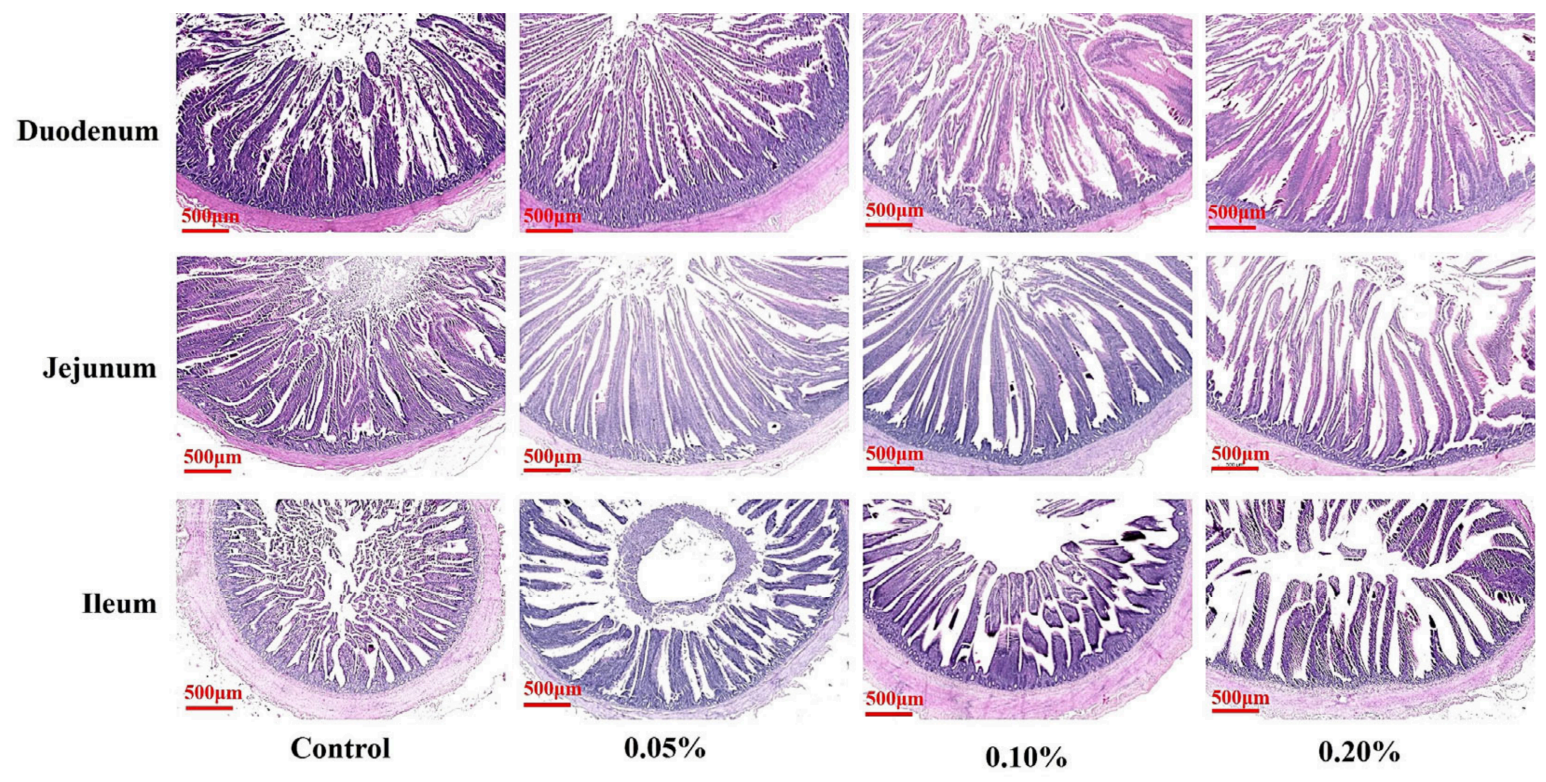
Histological micrographs of duodenal, jejunal and ileal tissues from laying hens fed PJP-supplemented diets. Micrograph magnification: 7 × 20.

VH, CD, and the V/C ratio serve as indicators to evaluate intestinal mucosal barrier integrity and general intestinal health. As illustrated in Table 6 and Figure 1, dietary supplementation with PJP significantly enhanced the duodenal mucosal morphology. Specifically, duodenal VH was markedly increased in the 0.05% and 0.20% supplementation groups (*p* < 0.05). Meanwhile, duodenal CD declined significantly within the 0.10% treatment cohort. The V/C ratio was significantly higher in the 0.05% and 0.10% groups, with the 0.10% group exhibiting the most favorable effect (*p* < 0.05). However, this beneficial effect on the mucosal structure was limited to the duodenum, and no such positive alterations were detected in the jejunum or ileum.

### 3.6. PJP Remodels the Cecal Microbial Community of Laying Hens

**Figure 2:**
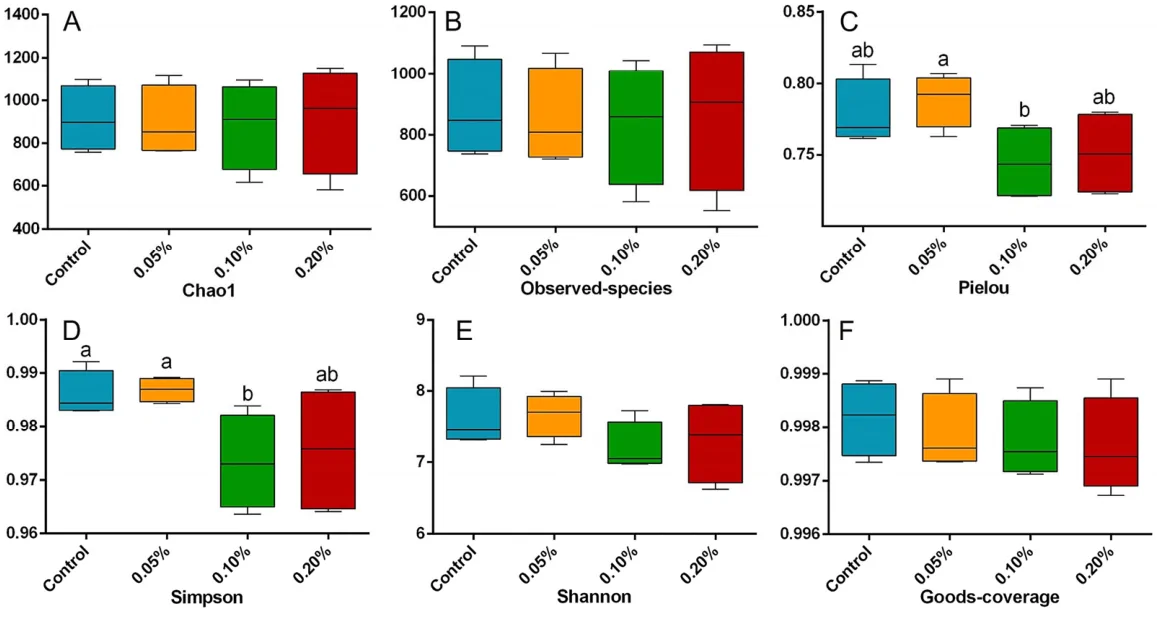
Alpha-diversity metrics of cecal microbial communities in laying hens fed PJP-supplemented diets. (**A**–**F**) are the Chao 1 index, Observed-species index, Pielou index, Simpson index, Shannon index and Goods-coverage index of intestinal microorganisms after PJP was added to the laying hens’ diet, respectively. Bars in blue, orange, green, and red represent the four experimental groups: the control diet (0.00% PJP), and basal diets supplemented with 0.05%, 0.10%, and 0.20% PJP, respectively. Different superscript letters in the same row indicate significant differences (*p* < 0.05).

To explore how dietary PJP inclusion shapes the diversity of cecal microbial communities, cecal digesta collected from laying hens were analyzed via 16S rRNA gene sequencing. As shown in Figure 2, the α-diversity analysis indicated that the Chao 1, Observed-species, Shannon, and Goods-coverage indices exhibited no notable alterations among dietary treatments with PJP inclusion (*p* > 0.05). However, variations in evenness were noted, as indicated by the Pielou index, which increased at the 0.05% supplementation level but decreased at the 0.10% and 0.20% levels. The 0.10% treatment displayed a significant drop in the Simpson index, reflecting shifts in community dominance (*p* < 0.05).

**Figure 3:**
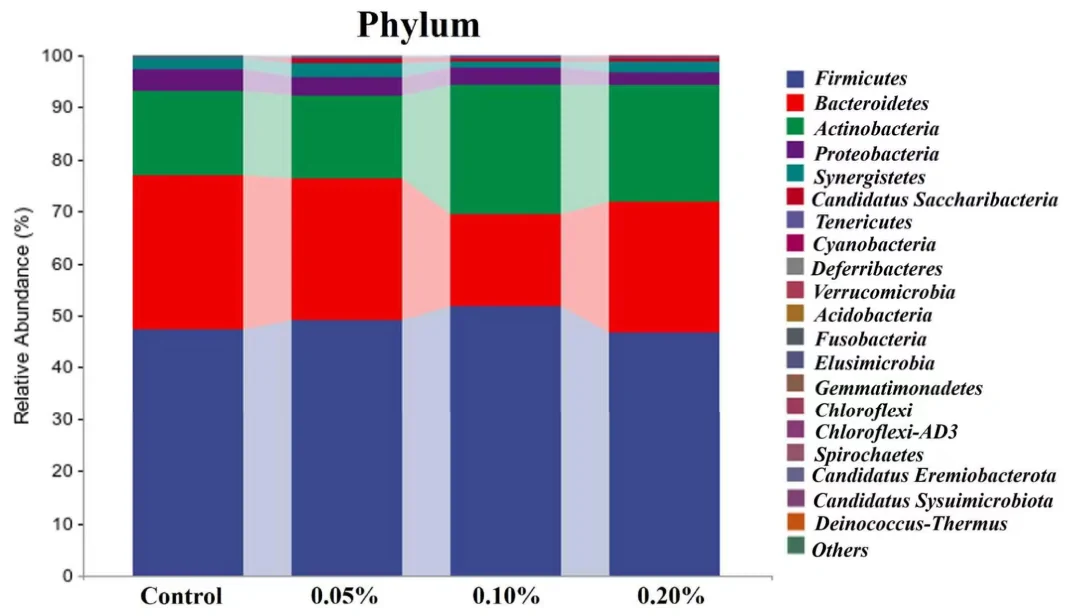
Taxonomic profiles at the phylum level for cecal microbiota in laying hens fed graded PJP diets.

**Figure 4:**
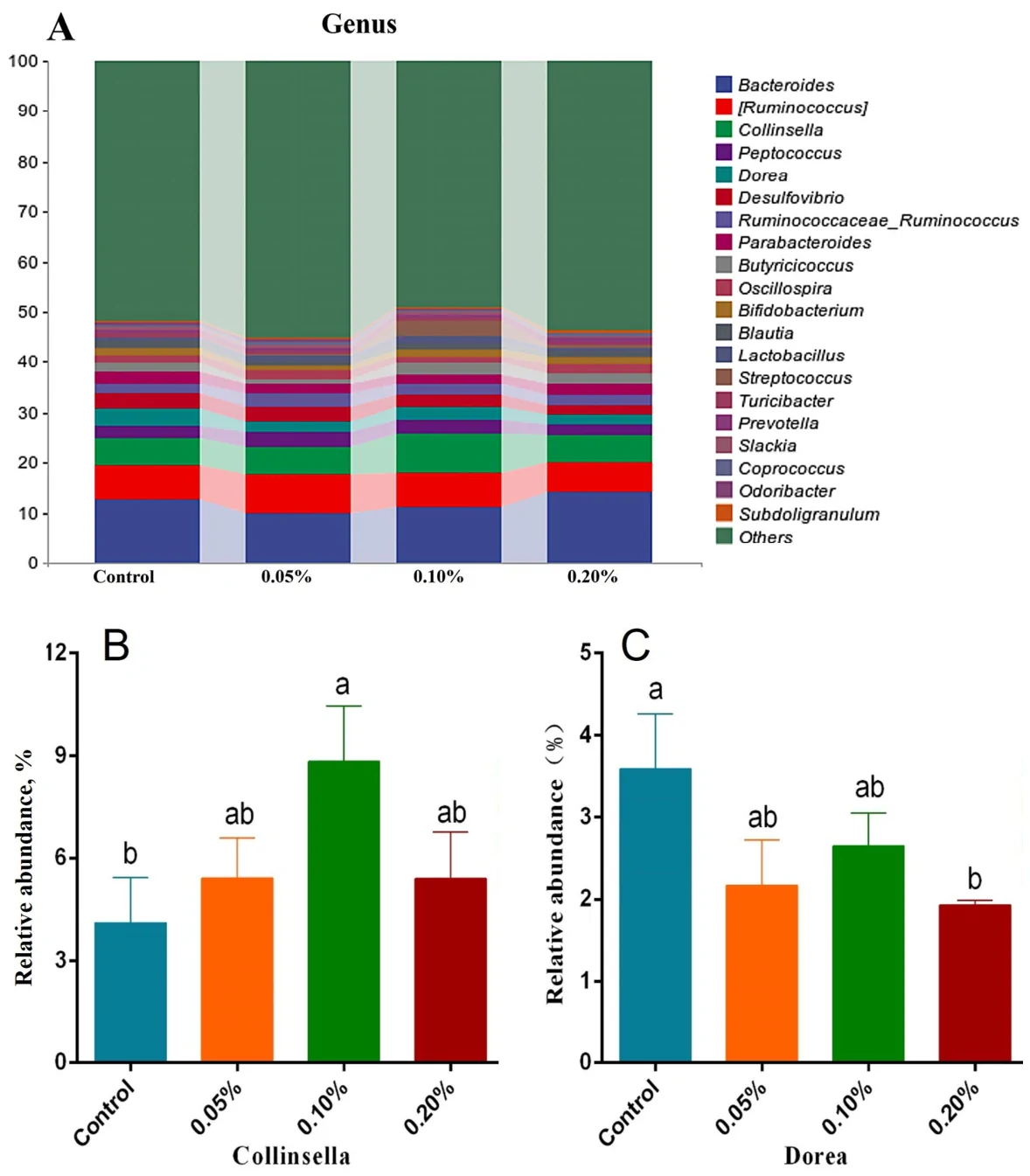
Genus-level taxonomic profiles of cecal microbiota in laying hens fed diets with graded PJP inclusion. (**A**) Profiles of predominant cecal genera in laying hens with different dietary PJP additions. (**B**) Relative abundance trends of *Collinsella*. (**C**) Relative abundance trends of *Dorea*. Different superscript letters in the same row indicated significant differences (*p* < 0.05). Bars in blue, orange, green, and red represent the four experimental groups: the control diet (0.00% PJP), and basal diets supplemented with 0.05%, 0.10%, and 0.20% PJP, respectively.

The composition of the cecal microbial community in response to PJP supplementation is illustrated in Figure 3. The dominant phyla identified were Firmicutes, Bacteroidetes, and Actinobacteria. Although not statistically significant, treatments with 0.10% and 0.20% PJP exhibited a trend towards increased abundances of Actinobacteria, while Bacteroidetes and Proteobacteria showed a decrease. Major bacterial genera within the cecal community included *Bacteroides*, *Ruminococcus*, *Collinsella*, and *Peptococcus*, as detailed in Figure 4A. Notably, the genus *Collinsella* became more prevalent in the cecal microbiota with supplementation, showing a marked elevation at the 0.10% inclusion level (Figure 4B). In contrast, the abundance of *Dorea* decreased across all treated groups, achieving statistical significance at the 0.20% supplementation level (*p* < 0.05, Figure 4C).

**Figure 5:**
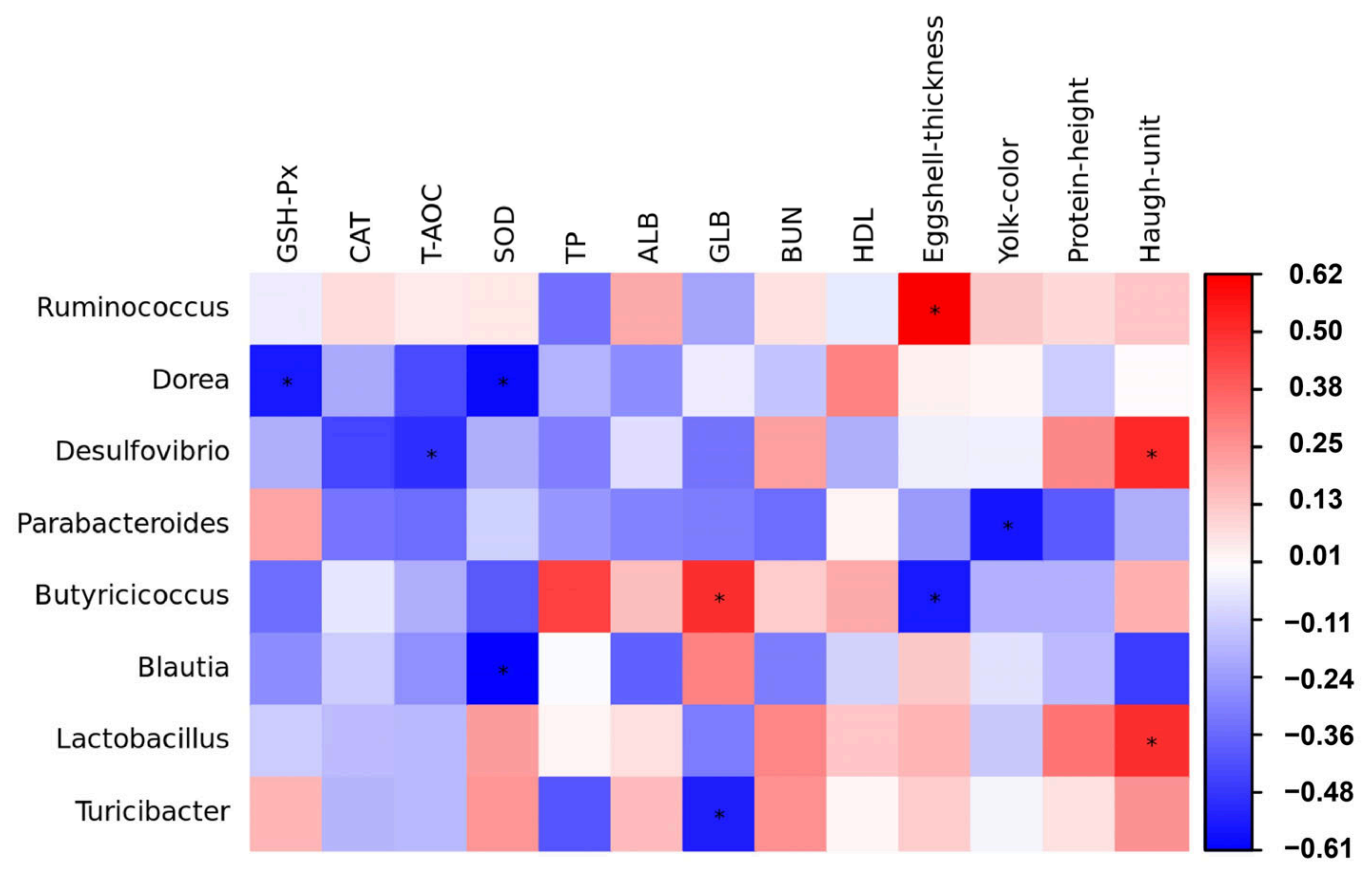
Spearman correlation heatmap linking cecal bacterial genera to antioxidant, serum biochemical and egg-quality traits. Red cells indicate positive correlations, whereas blue cells denote negative correlations. Color intensity corresponds to the magnitude of correlation coefficients. * *p* < 0.05.

Following the characterization of cecal microbial community profiles in laying hens, the top 20 genera in relative abundance were subjected to Spearman correlation analysis against antioxidant markers, serum protein parameters, and egg quality traits. As illustrated in Figure 5, eight genera exhibited significant correlations with the antioxidant capacity, protein levels, and egg quality of the laying hens. *Dorea* demonstrated significant negative correlations with GSH-Px and SOD activities. Similarly, *Desulfovibrio* showed a negative association with T-AOC activity, while *Blautia* was inversely related to SOD activity (*p* < 0.05). Ruminococcus displayed a strong positive correlation with eggshell thickness, but *Butyricicoccus* showed an inverse association with eggshell thickness, Parabacteroides exhibited a negative association with yolk color, and *Lactobacillus* and *Desulfovibrio* exhibited positive associations with the Haugh unit (*p* < 0.05).

## 4. Discussion

The significant increase in eggshell thickness at 0.05% PJP aligns with the capacity of several flavonoid-rich plant materials to improve eggshell quality in laying hens [15,16]. Because shell formation depends on mineral supply and shell-gland function, and a favorable antioxidative status within the uterus is thought to support calcium deposition [16], the antioxidant activity of PJP flavonoids may have supported shell-gland function at this moderate level; direct uterine and calcium-metabolism measurements are nevertheless required to confirm this. However, in this trial, albumen height and Haugh unit in the 0.20% treatment exhibited merely numerical declines relative to the basal control, yet these two indicators were significantly reduced compared with the 0.05% and 0.10% treatments *(p* < 0.05). In late-phase hens, oxidative stress and impaired reproductive function are considered to drive albumen dilution and the age-related decline in albumen height and Haugh unit [17], which moderate plant-derived supplementation has been reported to mitigate [18,19]. Accordingly, albumen quality was maintained at 0.05% and 0.10% PJP, whereas the significant reduction in the 0.20% group implies an upper inclusion boundary. This biphasic pattern is consistent with the view that polyphenols benefit performance only within a limited range and may become anti-nutritional at high levels through bile-salt binding and digestive-enzyme interference [20], and that egg-white quality often peaks at an intermediate dosage before decreasing [21]; even an otherwise antioxidant material, *Artemisia argyi*, was reported to turn pro-oxidative and disturb intestinal morphology at 3% inclusion [22]. Given that the secondary-metabolite content of PJP is expected to rise with dietary inclusion, the highest dosage may have impeded protein digestion or oviductal albumen synthesis. Overall, these results appear to favor a suitable, moderate inclusion range for PJP in late-phase layers rather than a linear benefit.

In the late laying cycle, laying hens display reduced tolerance against oxidative stress, and dietary provision of antioxidant compounds can strengthen the animals’ ability to scavenge free radicals and sustain redox homeostasis [23,24]. Consistent with this premise, PJP dietary inclusion in this trial raised the functional activities of key antioxidant enzymes (GSH-Px; CAT; SOD) together with hepatic T-AOC across all supplemented treatments relative to the control, thereby corroborating this antioxidant effect. Beyond this direct action, the improved antioxidant status appears to confer additional benefits. For instance, adequate antioxidant capacity in the shell gland might help to prevent oxidative damage and could support calcium deposition during eggshell calcification, possibly favoring eggshell quality [25]. Likewise, enhanced antioxidant capacity seems to contribute to reshaping the cecal microbial community in aged hens [26], which might be related to the increased eggshell thickness and the modulated cecal microbiota observed here. However, the highest hepatic antioxidant enzyme activities at 0.20% PJP coincided with the poorest albumen quality—an apparently paradoxical result. This discrepancy may be explained by the excessive antioxidant activity of PJP, which at high inclusion levels might simultaneously reduce nutrient digestibility and digestive enzyme activity despite strengthening hepatic antioxidant defenses [27,28].

The enzymes ALP, AST, and ALT provide critical diagnostic information regarding liver pathology, as elevations in these markers indicate hepatocyte damage, necrosis, and intrahepatic cholestasis [29]. The results indicated that the levels of these three hepatic enzymes remained comparable across all dietary treatments, suggesting that varying levels of PJP did not adversely affect the hepatic function of laying hens. When hepatic injury occurs, the protein synthesis capacity is impaired, leading to decreased levels of TP and ALB, whereas elevated levels indicate enhanced protein metabolism [30,31]. Within the context of this study, the increased TP and ALB levels may suggest that PJP could promote hepatic protein anabolism and enhance protein deposition in tissues, thereby potentially facilitating nutrient absorption and metabolism in laying hens.

Small intestinal morphology is a critical determinant of digestive and absorptive function, and V/C acts as a core metric for evaluating the structural integrity and physiological function of intestinal mucosal tissues [32]. In this study, PJP supplementation increased the duodenal V/C ratio, particularly at 0.05% and 0.10%, suggesting improved intestinal morphology. As villous architecture directly supports nutrient absorption and mucosal integrity [33], these changes, together with reduced crypt depth, may enhance nutrient assimilation. Such morphological improvements might reflect an accelerated renewal and increased maturation of intestinal epithelial cells, which could enlarge the intestinal absorption area, thereby facilitating nutrient uptake [34].

The cecal microbiota and the host co-evolve through a long-term symbiotic relationship, contributing to numerous host physiological processes [35]. Dietary composition is a major driver of gut microbial composition and host metabolic responses; such diet-induced remodeling is not confined to poultry, as comparable effects on intestinal development have also been observed in pigs—which may partly account for the PJP-induced shifts in cecal microbial community observed in the present study [36,37]. In the present study, the significantly decreased Simpson index (*p* < 0.05) in the 0.10% PJP group, despite unchanged species richness indices (Chao 1 and observed-species), may imply reduced community dominance and increased microbial diversity, which may be evidence of a more balanced microbial community [38,39]. Firmicutes, Bacteroidetes, and Actinobacteria remained the predominant phyla, consistent with profiles reported in healthy laying hens [8,40,41]. Notably, 0.10% PJP increased the relative abundance of *Collinsella*, a genus capable of synthesizing ursodeoxycholic acid (UDCA), a metabolite with reported antioxidant, anti-apoptotic, and anti-inflammatory activities [42]. It is therefore plausible that the enrichment of *Collinsella* partly contributed to the improved antioxidant status observed. Conversely, PJP reduced the abundance of *Dorea*, a genus associated with hepatic steatosis, NASH, obesity, and pro-inflammatory conditions [43,44]; in light of the anti-inflammatory actions reported for plant-derived compounds [45], this reduction may suggest a milder intestinal inflammatory state, although such a possibility remains speculative because inflammatory mediators were not directly measured in the present study. Plant-derived feed additives that enrich beneficial genera such as *Lactobacillus* and *Ruminococcus* have been shown to concurrently improve eggshell thickness and the activities of antioxidant enzymes (e.g., catalase, CAT, and glutathione peroxidase, GSH-Px) while reducing malondialdehyde [46], and the increased abundance of *Ruminococcus* has been associated with enhanced nutrient digestibility in laying hens [47]. In line with the above evidence, the current Spearman correlation analysis demonstrated that *Ruminococcus* and *Lactobacillus* were positively correlated with the eggshell thickness and Haugh unit, respectively, whereas *Dorea*, *Desulfovibrio*, and *Blautia* showed negative correlations with antioxidant indices [48]. Collectively, PJP supplementation enhanced antioxidant capacity and enriched beneficial cecal taxa; these improvements may act in concert to support calcium metabolism and nutrient assimilation, plausibly contributing to the improved eggshell thickness observed in this study, while the exact causal mechanisms behind these phenotypic variations still require further systematic verification.

Overall, the 0.10% PJP group exhibited the most balanced improvements across all measured endpoints: it enhanced hepatic antioxidant capacity, most effectively improved the duodenal V/C ratio, and favorably modulated the cecal microbiota, while egg quality was maintained and no adverse effects were observed. In contrast, although antioxidant enzyme activities were highest in the 0.20% supplementation group, albumen quality was lower than that of the control group, and the 0.05% supplementation group significantly increased eggshell thickness yet exerted weaker improvements in intestinal morphology and microbial diversity relative to the 0.10% group. Considering the net benefits across antioxidant capacity, intestinal morphology, cecal microbiota, and egg quality, 0.10% PJP was therefore recommended as the optimal inclusion level under the conditions of this study.

A limitation of the present study is that a formal safety assessment of PJP was not performed. Nevertheless, no mortalities, clinical signs of poisoning, or adverse changes in hepatic enzyme activities (AST, ALT, and ALP) were observed in any dietary treatment during either the 2-week preliminary phase or the 8-week formal experimental period, indirectly suggesting that the 0–0.20% inclusion range tested was well tolerated. However, because the concentration of potentially bioactive compounds in PJP was not determined, potential risks at higher doses or with prolonged feeding cannot be fully excluded. Future studies should therefore quantify these compounds in PJP and further evaluate the safe dietary threshold.

## 5. Conclusions

In summary, dietary PJP inclusion may improve the eggshell thickness of eggs from laying hens by enhancing host antioxidant defense, reshaping the composition of cecal microbial communities. Based on observations from this trial, 0.10% is suggested as the appropriate amount of PJP to include in these hens’ diet.

## Figures and Tables

**Table 1:** Ingredient constituents and nutritional profiles of the basal experimental diets (air-dry basis), %.

Items	Content (%)
Ingredients	
Corn	57.57
Soybean meal	30.01
Soybean oil	0.97
Limestone	8.45
3% Premix ^1^	3.00
Total	100.00
Calculated nutrient levels	
Metabolizable energy (Mcal/kg)	2.75
Crude protein	17.00
Crude fiber	3.04
Calcium	3.50
Total phosphorus	0.54
Available phosphorus	0.32
Digestible lysine	0.66
Digestible methionine	0.36
Digestible threonine	0.56
Digestible tryptophan	0.15

Note: The values represent the basal control diet. Given the low supplemental dosages of PJP (maximum 0.20%), PJP was top-dressed directly into the complete basal diet without replacing any feed ingredients. Thus, the proportions of corn, soybean meal, soybean oil, limestone and premix remained identical across all four dietary treatments. The small addition rate of PJP exerts negligible influence on overall dietary nutrient composition. ^1^ The basal diet was supplemented with a vitamin–mineral premix containing (per kg of diet): vitamin A, 6000 IU; vitamin D_3_, 2500 IU; vitamin E, 25 mg; vitamin K_3_, 2.25 mg; vitamin B_1_, 1.8 mg; vitamin B_2_, 7 mg; vitamin B_6_, 4 mg; vitamin B_12_, 0.2 mg; D-pantothenic acid, 12 mg; nicotinic acid, 35 mg; biotin, 0.14 mg; folic acid, 0.8 mg; copper (from CuSO_4_), 11 mg; zinc (from ZnSO_4_), 70 mg; iron (from FeSO_4_), 60 mg; manganese (from MnSO_4_), 115 mg; selenium (from Na_2_SeO_3_), 0.30 mg; and iodine (from KI), 0.4 mg.

**Table 2:** Productive-performance responses of laying hens to graded dietary inclusion rates of PJP.

Items	Control	Addition Level of PJP			*p*-Value
		0.05%	0.10%	0.20%	
Daily feed intake, g/d	107.067 ± 2.975	108.900 ± 2.255	107.700 ± 1.363	108.400 ± 2.086	0.943
Egg weight, g	58.816 ± 0.251	58.484 ± 0.195	58.576 ± 0.265	58.686 ± 0.192	0.759
FCR, g/g	1.820 ± 0.049	1.862 ± 0.037	1.839 ± 0.021	1.847 ± 0.037	0.886
Egg-breaking rate, %	0.372 ± 0.075	0.591 ± 0.266	0.506 ± 0.328	0.000 ± 0.000	0.250 *
Egg-laying rate, %	88.624 ± 1.619	89.220 ± 1.805	88.889 ± 1.760	84.921 ± 1.778	0.292

Notes: * Egg-breaking rate contained zero-inflated data; the reported *p*-value should be interpreted with caution. Control = non-supplemented basal diet; 0.05% = basal diet incorporated with 0.05% PJP; 0.10% = basal diet incorporated with 0.10% PJP; 0.20% = basal diet incorporated with 0.20% PJP.

**Table 3:** Egg quality traits of laying hens fed diets containing graded inclusion levels of PJP.

Items	Control	Addition Level of PJP			*p*-Value
		0.05%	0.10%	0.20%	
Average egg weight, g	56.541 ± 0.844	57.301 ± 0.837	57.695 ± 1.043	58.948 ± 1.043	0.296
Shell weight/egg weight, %	10.102 ± 0.210	10.076 ± 0.175	10.092 ± 0.127	10.003 ± 0.127	0.972
Yolk weight/egg weight, %	28.464 ± 0.332	27.688 ± 0.594	27.340 ± 0.397	28.223 ± 0.490	0.348
Albumen weight/egg weight, %	61.434 ± 0.499	62.237 ± 0.738	62.568 ± 0.448	61.774 ± 0.477	0.516
Egg shape index, %	76.507 ± 0.984	76.995 ± 0.997	77.527 ± 1.312	78.288 ± 1.141	0.780
Eggshell thickness, mm	0.386 ± 0.004 ^b^	0.419 ± 0.011 ^a^	0.386 ± 0.003 ^b^	0.384 ± 0.004 ^b^	0.001
Yolk color	6.546 ± 0.157 ^ab^	6.917 ± 0.193 ^a^	6.182 ± 0.122 ^b^	6.500 ± 0.151 ^ab^	0.022
Albumen height, mm	5.155 ± 0.164 ^ab^	5.942 ± 0.197 ^a^	5.545 ± 0.355 ^a^	4.658 ± 0.334 ^b^	0.012
Haugh unit	71.000 ± 1.427 ^ab^	76.917 ± 1.345 ^a^	72.901 ± 3.046 ^a^	64.500 ± 3.098 ^b^	0.005

Note: Values within the same row bearing distinct superscript letters differ significantly (*p* < 0.05). Control = non-supplemented basal diet; 0.05% = basal diet incorporated with 0.05% PJP; 0.10% = basal diet incorporated with 0.10% PJP; 0.20% = basal diet incorporated with 0.20% PJP.

**Table 4:** Effects of PJP on hepatic antioxidant indices of laying hens.

Items	Control	Addition Level of PJP			*p*-Value
		0.05%	0.10%	0.20%	
GSH, mg/L	6.925 ± 0.765	9.428 ± 1.794	7.018 ± 1.036	11.277 ± 3.185	0.347
CAT, U/mL	4.975 ± 0.902 ^b^	7.558 ± 1.61 ^ab^	6.20 ± 1.14 ^b^	11.32 ± 1.89 ^a^	0.023
SOD, U/mL	111.954 ± 10.647 ^b^	142.052 ± 5.850 ^a^	127.400 ± 5.671 ^ab^	145.220 ± 6.742 ^a^	0.022
GSH-Px, μmol/L	478.018 ± 45.272 ^b^	573.620 ± 25.240 ^ab^	539.902 ± 16.730 ^ab^	629.752 ± 34.339 ^a^	0.027
T-AOC, U/mL	0.486 ± 0.039 ^b^	0.698 ± 0.049 ^a^	0.655 ± 0.031 ^a^	0.772 ± 0.071 ^a^	0.008
MDA, nmol/mL	31.783 ± 11.460	20.857 ± 4.944	19.443 ± 2.503	12.332 ± 2.626	0.282

Note: Values within the same row bearing distinct superscript letters differ significantly (*p* < 0.05). Control = non-supplemented basal diet; 0.05% = basal diet incorporated with 0.05% PJP; 0.10% = basal diet incorporated with 0.10% PJP; 0.20% = basal diet incorporated with 0.20% PJP.

**Table 5:** Serum biochemical marker profiles of laying hens fed diets supplemented with PJP.

Items	Control	Addition Level of PJP			*p*-Value
		0.05%	0.10%	0.20%	
TP, g/L	55.914 ± 1.478 ^b^	61.360 ± 2.161 ^ab^	67.836 ± 3.826 ^a^	62.456 ± 1.942 ^ab^	0.032
ALB, g/L	20.147 ± 0.590 ^b^	22.226 ± 0.972 ^ab^	23.828 ± 0.836 ^a^	20.696 ± 0.572 ^b^	0.012
GLB, g/L	35.844 ± 1.133 ^b^	39.134 ± 1.457 ^ab^	44.516 ± 2.680 ^a^	39.625 ± 1.678 ^ab^	0.030
ALB/GLB	0.562 ± 0.024	0.570 ± 0.022	0.524 ± 0.018	0.538 ± 0.033	0.501
BUN, mmol/L	0.280 ± 0.037 ^b^	0.337 ± 0.038 ^ab^	0.385 ± 0.021 ^a^	0.257 ± 0.021 ^b^	0.032
AST, U/L	293.857 ± 29.775	273.113 ± 16.93	296.567 ± 10.812	288.17 ± 32.159	0.888
ALT, U/L	5.900 ± 1.254	6.055 ± 1.500	9.327 ± 1.577	7.318 ± 1.570	0.356
AST/ALT	42.582 ± 6.750	58.775 ± 12.501	26.075 ± 1.980	58.806 ± 16.165	0.219
ALP, U/L	375.226 ± 49.151	234.478 ± 33.491	479.095 ± 69.630	387.193 ± 79.486	0.098
LDH, U/L	934.833 ± 50.234	943.667 ± 74.255	1132.500 ± 207.030	846.333 ± 153.022	0.519
GLU, mmol/L	12.496 ± 0.137	11.930 ± 0.51	13.335 ± 0.459	13.1121 ± 0.651	0.179
TC, mmol/L	3.078 ± 0.514	3.107 ± 0.308	3.008 ± 0.280	2.288 ± 0.255	0.197
TG, mmol/L	14.665 ± 2.446	13.165 ± 1.521	14.582 ± 2.315	13.022 ± 3.901	0.954
HDL, mmol/L	0.305 ± 0.038 ^a^	0.153 ± 0.023 ^b^	0.322 ± 0.022 ^a^	0.190 ± 0.042 ^b^	0.003
LDL, mmol/L	0.165 ± 0.038	0.140 ± 0.038	0.130 ± 0.020	0.153 ± 0.011	0.841

Note: Values within the same row bearing distinct superscript letters differ significantly (*p* < 0.05). Control = non-supplemented basal diet; 0.05% = basal diet incorporated with 0.05% PJP; 0.10% = basal diet incorporated with 0.10% PJP; 0.20% = basal diet incorporated with 0.20% PJP.

**Table 6:** Morphometric parameters of intestinal tissues in laying hens with PJP dietary inclusion.

Items	Control	Addition Level of PJP			*p*-Value
		0.05%	0.10%	0.20%	
Duodenal VH, μm	1128.932 ± 93.809 ^c^	1678.753 ± 172.038 ^ab^	1419.033 ± 17.011 ^bc^	2010.007 ± 76.544 ^a^	0.005
Duodenal CD, μm	257.910 ± 37.446 ^a^	213.517 ± 10.322 ^ab^	160.064 ± 0.461 ^b^	300.838 ± 23.592 ^a^	0.035
Duodenal V/C	4.483 ± 0.371 ^b^	7.970 ± 1.133 ^a^	8.875 ± 0.124 ^a^	6.721 ± 0.288 ^ab^	0.014
Jejunal VH, μm	1025.887 ± 26.05	1100.320 ± 211.085	1201.684 ± 22.352	1357.971 ± 42.147	0.290
Jejunal CD, μm	159.404 ± 1.511	147.952 ± 5.070	178.019 ± 2.731	185.802 ± 34.009	0.308
Jejunal V/C	6.443 ± 0.224	7.363 ± 1.145	6.751 ± 0.022	7.903 ± 1.645	0.791
Ileal VH, μm	856.365 ± 41.580	722.341 ± 55.677	891.605 ± 31.325	876.693 ± 3.402	0.058
Ileal CD, μm	166.984 ± 4.366	139.860 ± 8.124	139.079 ± 4.762	119.104 ± 21.415	0.144
Ileal V/C	5.137 ± 0.334	5.163 ± 0.231	6.430 ± 0.342	7.781 ± 1.175	0.057

Note: Values within the same row bearing distinct superscript letters differ significantly (*p* < 0.05). Control = non-supplemented basal diet; 0.05% = basal diet incorporated with 0.05% PJP; 0.10% = basal diet incorporated with 0.10% PJP; 0.20% = basal diet incorporated with 0.20% PJP.

## Data Availability

The original contributions presented in this study are included in the article/Supplementary Material. Further inquiries can be directed to the corresponding authors.

## Supplementary Materials

The following supporting information can be downloaded at https://www.mdpi.com/article/10.3390/vetsci13101116/s1: Table S1: Alpha diversity indices of cecal microbiota in each group; Table S2: Number of sequencing reads per sample; Table S3: Filtered ASV-table; Table S4: Taxonomic annotation results of ASVs at different taxonomic levels.
